# Adaptive response in human blood lymphocytes exposed to non-ionizing radiofrequency fields: resistance to ionizing radiation-induced damage

**DOI:** 10.1093/jrr/rrt106

**Published:** 2013-08-26

**Authors:** Anna Sannino, Olga Zeni, Stefania Romeo, Rita Massa, Giancarlo Gialanella, Gianfranco Grossi, Lorenzo Manti, Vijayalaxmi, Maria Rosaria Scarfì

**Affiliations:** 1 CNR – Institute for Electromagnetic Sensing of the Environment, via Diocleziano 328, 80124, Napoli, Italy; 2 National Institute of Nuclear Physics, Section of Napoli, via Cintia, 80126, Napoli, Italy; 3 Department of Physics, University of Naples Federico II, CMSA via Cintia, 80126, Napoli, Italy; 4 Centre of Radioprotection and Health Physics, University of Naples Federico II, via Cintia, 80126, Napoli, Italy; 5 Department of Radiology, University of Texas Health Science Centre, 7703 Floyd Curl Drive – MC 7800, San Antonio, TX 78229-3900, USA

**Keywords:** radiofrequency, adaptive response, micronucleus, X-rays, human lymphocytes

## Abstract

The aim of this preliminary investigation was to assess whether human peripheral blood lymphocytes which have been pre-exposed to non-ionizing radiofrequency fields exhibit an adaptive response (AR) by resisting the induction of genetic damage from subsequent exposure to ionizing radiation. Peripheral blood lymphocytes from four healthy donors were stimulated with phytohemagglutinin for 24 h and then exposed for 20 h to 1950 MHz radiofrequency fields (RF, adaptive dose, AD) at an average specific absorption rate of 0.3 W/kg. At 48 h, the cells were subjected to a challenge dose (CD) of 1.0 or 1.5 Gy X-irradiation (XR, challenge dose, CD). After a 72 h total culture period, cells were collected to examine the incidence of micronuclei (MN). There was a significant decrease in the number of MN in lymphocytes exposed to RF + XR (AD + CD) as compared with those subjected to XR alone (CD). These observations thus suggested a RF-induced AR and induction of resistance to subsequent damage from XR. There was variability between the donors in RF-induced AR. The data reported in our earlier investigations also indicated a similar induction of AR in human blood lymphocytes that had been pre-exposed to RF (AD) and subsequently treated with a chemical mutagen, mitomycin C (CD). Since XR and mitomycin-C induce different kinds of lesions in cellular DNA, further studies are required to understand the mechanism(s) involved in the RF-induced adaptive response.

## INTRODUCTION

The phenomenon of adaptive response (AR) was first demonstrated in human peripheral blood lymphocytes by Olivieri *et al*. [1], i.e. cells which had been pre-exposed to a very low dose of ionizing radiation (adaptive dose, AD) were resistant to the genetic damage induced by subsequent exposure to a high genotoxic dose of ionizing radiation (challenge dose, CD). Several subsequent reports in human and animal cells have confirmed the existence of AR, and resistance not only to the same but also cross-resistance to similar genotoxic agents. Further investigations have also characterized ionizing radiation-induced AR [2–14].

Non-ionizing radiofrequency fields (RFs), in the 800–3000 MHz frequency range, have been extensively used in wireless communication systems that deliver voice, data and images. Over recent decades, several researchers have exposed freshly collected and cultured mammalian cells to different RF frequencies, modulations, specific absorption rate (SAR), exposure durations, etc. to investigate whether or not such RF exposure induces excess genotoxicity evaluated from DNA strand breaks, chromosomal aberrations, micronuclei, sister chromatid exchanges and mutations. Investigations are also being conducted to examine the impact of simultaneous/sequential exposure of RF with other physical/chemical genotoxic agents. Several reviews have already been published, the conclusions being that RF exposure alone has no genotoxic potential while the combined exposure effects need further investigation [see recent reviews in 15, 16].

Our research group demonstrated for the first time that pre-exposure of human cells to non-ionizing RF (AD), non-genotoxic *per se*, is capable of inducing an AR by eliciting resistance to damage induced by subsequent exposure to known genotoxic agents (CD). Our results indicated that human blood lymphocytes that had been pre-exposed to 900 MHz RF, GSM modulation at an average SAR of 1.25 W/kg, exhibited a significantly decreased incidence of micronuclei (MN) when subsequently treated with a genotoxic dose of mitomycin C (MMC) [17], suggesting an RF-induced AR. These observations were confirmed in two subsequent investigations [18, 19]. Similarly, an RF-induced AR was also reported by independent research groups in experimental mice, rats and continuously growing cultured human cells [20–25].

So far, we have examined an RF-induced AR using MMC (as CD), an alkylating chemical mutagen which induces cross-links and causes DNA damage. The current study was aimed to investigate whether human blood lymphocytes which had been pre-exposed to RF exhibit resistance to the genetic damage induced by subsequent exposure to X-rays (XR), an ionizing physical mutagen that induces predominantly strand breaks in DNA. The genetic damage is assessed using the classical MN assay and the results are discussed below.

## MATERIALS AND METHODS

All reagents were purchased from BioWhittaker (Verviers, Belgium), except for L-glutamine (Gibco, Milan, Italy) and MMC (Sigma, St Louis, MO). The same batch of reagents was used for all experiments, which were performed ‘blind’ such that the person evaluating the extent of genetic damage was not aware of the exposure conditions.

### RF/sham exposures and dosimetry

**Fig. 1.**
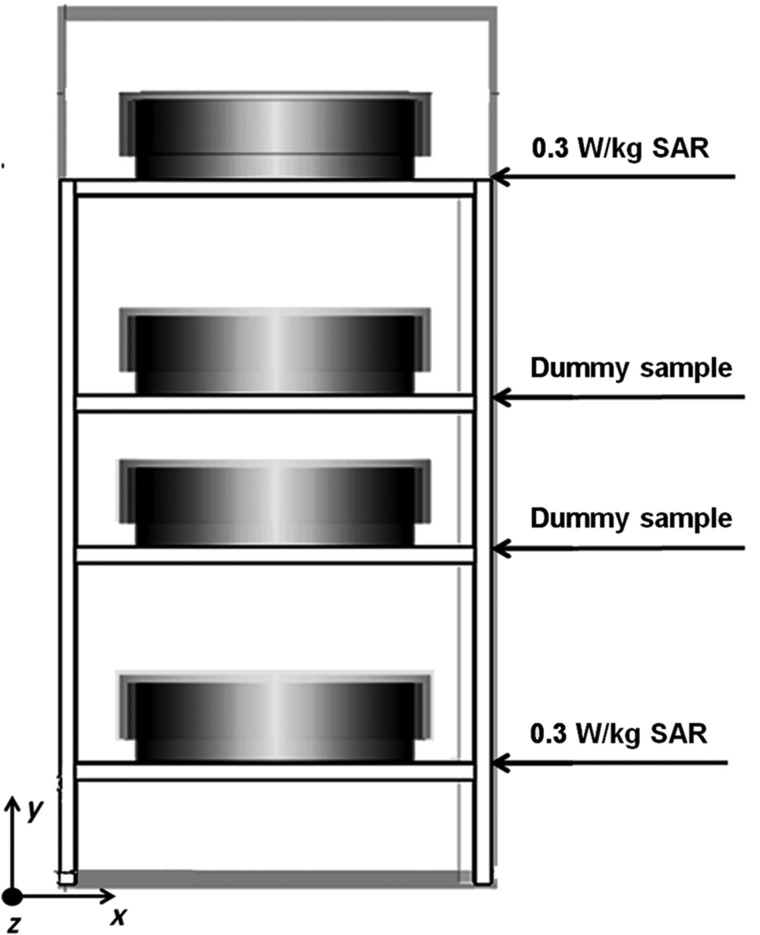
Arrangement of Petri dishes in the waveguide. Lymphocytes cultures in Petri dishes are kept in the outer positions (0.3 W/kg SAR), while dummy samples are located in the inner ones.

A short-circuited waveguide-based exposure system was employed and described in great detail in our earlier publication [26]. It was originally designed to expose four cell cultures in 35-mm Petri dishes to two different SAR levels simultaneously, by placing sample centres in a specific position inside the waveguide and at a specific distance from each other. In such configuration, when RF was transmitted, the SAR for cell cultures kept in outer positions was one-quarter that of cell cultures kept in inner positions. In this study, the Petri dishes containing the diluted blood from each donor (see below) were kept in the outer positions and exposed to 0.3 W/kg, while dummy samples were placed in the inner positions (Fig. 1). The cells were allowed to settle at the bottom before subjecting them to RF and sham exposures.

**Fig. 2.**
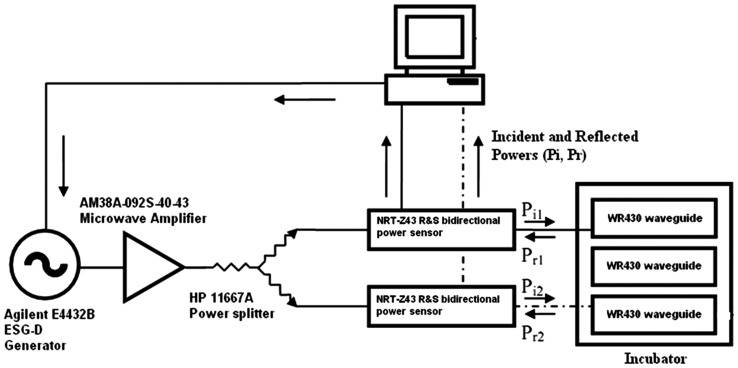
RF exposure set up. Schematic presentation of the set-up used to expose the cells to 1950 MHz (UMTS signal) radiofrequency field/sham. The signal generator and power sensors are connected to a computer installed with home-made software for continuous monitoring and recording of the power level as it was adjusted to achieve the required 0.3 W/kg SAR.

The 1950-MHz RF UMTS (Universal Mobile Telecommunication Systems) signal was generated by an E4432B ESG-D series generator (Agilent, Santa Clara, California), amplified (Microwave Amplifiers, Ltd AM38A-092 S-40–43, North Somerset, Bristol, UK), split (11667A power splitter, Hewlett-Packard, Palo Alto, CA, USA) and fed into twin exposure chambers through bidirectional NRT-Z43 power sensors (Rohde and Schwarz, Munich, Germany). The generator and the power sensors were connected to a computer with home-made software that controlled the power level required to expose the cells at an average SAR of 0.3 W/kg. The sham exposures were conducted in an identical waveguide without RF transmission. Both RF and sham rectangular short-circuited waveguides (Sairem, Neyron, France; 54.6 width × 109.2 mm height) with coaxial adapters (Maury Microwave R213A2, VSWR:1.05, Mont Clair, California) at the feeding end were placed in a commercial incubator (model 311, Forma Scientific, Freehold, NJ, USA) maintaining 37.0 ± 0.5°C temperature and a humidified atmosphere of 5% carbon dioxide and 95% air, which ensured controlled exposure as well as controlled cell culture conditions. The RF exposure set-up is schematically presented in Fig. 2. A detailed and realistic numerical dosimetry was carried out using a CST-Microwave Studio (Darmstadt, Germany), which considered the stratification/sedimentation of the cells, dielectric properties of the blood, and the relative permittivity and conductivity of the RPMI medium [19]. The average SAR obtained was 0.3 W/kg with uniform distribution (coefficient of variation<33%).

Temperature measurements were also carried out at regular 5-s intervals for 20 h (accuracy of ± 0.3°C) in a separate experiment under the same experimental RF-exposure, using a fiber-optic thermometer (FisoUMI4, FISO Technologies, Quebec, Canada) with a fiber-optic temperature probe (FISO Technologies, FOT-M/2 m) inserted horizontally into the culture medium. Five independent measurements demonstrated that the temperature never exceeded the instrument sensitivity (± 0.3°C).

### X-irradiation

The X-ray machine (Siemens Stabiliplan, Germany; 120-kVp, 20 mA half value layer, 4.0 mm Al) located at the Centre of Radioprotection and Health Physics, University of Naples Federico II (Napoli, Italy) was used for X-irradiation of the cells. The dose rates used to expose the cells to 1.0 cGy and 1.0 or 1.5 Gy were 0.045 Gy/min and 0.75 Gy/min, respectively. The dose was checked and controlled during irradiation using a cylindrical ionization chamber (N30001 0.6 cm^3^ acrylic/graphite PTW Farmer, Freiburg, Germany) connected to an electrometer (37-725 Victoreen, Cleveland, USA): the point of measurement in the ion chamber was located near the Petri dishes. Four Petri dishes were irradiated at a time, and inhomogeneity of the dose within the radiation field was 2%.

### Experimental protocol

The ethical committee of San Paolo Hospital in Naples (ASL Napoli 1) approved all of the experimental procedures used in this investigation. Written informed consent was obtained from the donors, following the internal rules of the hospital. Blood samples from four healthy, non-smoking male donors—D1, D2, D3 and D4—between 23 and 36 years of age were collected in sterile, heparinized vacutainer tubes (Becton Dickinson, NJ, USA).

**Fig. 3.**
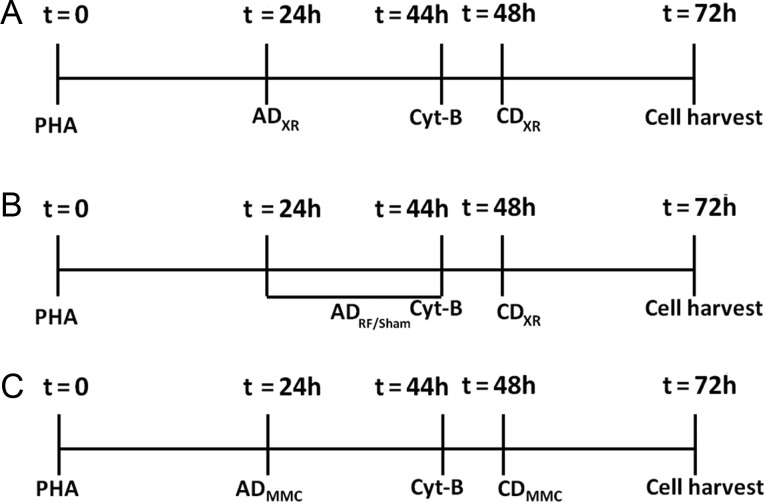
Experimental protocols. Panel **A**: exposure of cultures to X-rays (XR). AD_XR_: adaptive dose of 1.0 cGy XR. CD_XR_: challenge dose of 1.0 or 1.5 Gy XR; Panel **B**: exposure of RF/sham experimental cultures. AD_RF_: adaptive dose of 1950 MHz RF at 0.3 W/kg SAR. CD_XR_: challenge dose of 1.0 or 1.5 Gy XR; Panel **C**: treatment of cultures using MMC. AD_MMC_: adaptive dose of 1 ng/ml MMC. CD_MMC_: challenge dose of 100 ng/ml MMC.

The blood was diluted (1:10) with RPMI 1640 culture medium containing 15% heat-inactivated fetal bovine serum, 1% phytohemagglutinin (PHA), 2 mM L-glutamine, 100 U/ml penicillin and 100 mg/ml streptomycin. Separate cultures were set up, each containing 3 ml of diluted blood in 35-mm Petri-dishes (Corning, New York, USA) and kept for 24 h in a cell culture incubator (37.0 ± 0.5°C, 95% air, 5% carbon dioxide). Then, the cells were exposed to: (i) 1 cGy XR (AD) followed by exposure to 1.0 Gy or 1.5 Gy XR (CD) at 48 h (Fig. 3A) (this experimental protocol was to determine whether AR can be observed in cells exposed to both low and high doses of XR), and (ii) RF/sham for 20 h (AD) followed by exposure to 1.0 Gy or 1.5 Gy XR (CD) at 48 h (Fig. 3B) (this experimental protocol was to determine whether the cells exposed to RF/sham and a high dose of XR show a significantly decreased MN frequency induced by CD, and thus exhibit RF-induced AR). Furthermore, (iii) a set of cell cultures was also exposed to 1 ng/ml MMC (AD) followed by exposure to 100 ng/ml MMC (CD) at 48 h (Fig. 3C) (this experimental protocol was similar to that used in our previous studies to confirm the induction of AR in cells exposed to both low and high doses of MMC).

### Cytokinesis-block micronucleus assay and data collection

At 44 h after PHA stimulation, cytochalasin B (6 µg/ml, Sigma, St Louis, MO, USA) was added to all cultures. At the end of 72 h total culture period, cells were harvested on microscope slides using a cytospin centrifuge (Shandon, Thermo Scientific, Pitsburgh, PA, USA). The slides were then fixed in 80% methanol and stained with 10% Giemsa solution. Coded slides were examined at × 1000 magnification with a light microscope (Dialux 22, Leica Microsystems, Mannheim, Germany). For each donor and from each exposure condition: (i) the extent of genotoxicity was assessed from the incidence of MN which was recorded in a total of 2000 consecutive binucleate cells (BN lymphocytes, 1000 each from duplicate slides) [27, 28], and (ii) 1000 cells were examined to calculate the proliferation index (PI), which was derived from [M1 + 2M2 + 3(M3 + M4)]/N, where M1 to M4 represent the numbers of cells with one to four nuclei, respectively, and N is the total number of cells scored [29]. The PI was taken as a measure of cytotoxicity in each exposure condition.

### Statistical analyses

The SAS software system for Windows, version 9.2.3 [30] was used to analyze all data. For each donor, the expected frequencies of MN in combined treatments (AD + CD) were calculated as the sum of the MN in two individual treatments minus the frequency in untreated control cells, as in our earlier publications and also in the great majority of AR investigations [31, 32]. The statistical significance of the observed frequencies relative to the expected indices was evaluated for individual donors with the one-tailed *z*-test, and for all donors by means of analysis of variance (ANOVA) for mixed models, taking into account the repeated measures of treatments for each donor. Pair-wise comparison of the results from all donors between RF-exposed and the corresponding sham-exposed cells was also undertaken using paired two-tailed *t*-tests, with the corresponding results from ANOVA. The assumptions of equal variance and normal distribution were examined and improved with the standard method of log transformation of the incidence of MN as demonstrated by the skewedness, kurtosis, Shapiro–Wilks test of normality and scatter plot of residuals vs predicted values [33]. The paired two-tailed *t-*test was applied to analyze the results from PI.

## RESULTS

The incidences of MN in 2000 BN lymphocytes from each donor, and the average obtained for all donors, are presented in Table 1.

**Table 1.** Incidence of micronuclei in 2000 binucleate lymphocytes from four donors (D1–D4) exposed to 1.0 cGy ± 1.0 or 1.5 Gy XR, and 1950 MHz RF/sham ± 1.0 or 1.5 Gy XR, and to 1 ng/ml ± 100 ng/ml MMC

Treatment	Micronuclei/2000 binucleate lymphocytes				
	D1	D2	D3	D4	Average
1.0 cGy XR (AD)	12	18	10	7	12
1.0 Gy XR (CD)	96	197	155	118	141
1.5 Gy XR (CD)	141	210	243	150	186
1.0 cGy + 1.0 Gy XR (AD + CD)	87	107	163	96	113
1.0 cGy + 1.5 Gy XR (AD + CD)	90	141	188	48	117
Expected values (AD + 1.0 Gy)	95	202	156	121	
% decrease (AD + 1.0 Gy)	8.4	47.0	−4.5	20.7	
^*a*^*P-*value (AD + 1.0 Gy)	0.336	0.001	0.674	0.037	
Expected values (AD + 1.5 Gy)	140	215	244	153	
% decrease (AD + 1.5 Gy)	35.7	34.4	22.9	68.6	
^*a*^*P-*value (AD + 1.5 Gy)	0.001	0.001	0.003	0.001	
1950 MHz RF (AD)	10	18	8	4	10
1950 MHz RF + 1.0 Gy XR (AD + CD)	92	142	169	81	121
1950 MHz RF + 1.5 Gy XR (AD + CD)	95	149	236	60	135^*b*^
Expected values (AD + 1.0 Gy)	93	202	154	118	
% decrease (AD + 1.0 Gy)	1.1	29.7	−9.7	31.4	
^*a*^*P-*value (AD + 1.0 Gy)	0.5	0.006	0.81	0.005	
Expected values (AD + 1.5 Gy)	138	215	242	150	
% decrease (AD + 1.5 Gy)	31.2	30.7	2.5	60	
^*a*^*P-*value (AD + 1.5 Gy)	0.003	0.001	0.352	0.001	
Sham (AD)	10	15	9	5	10
Sham + 1.0 Gy XR (AD + CD)	82	184	153	110	132
Sham + 1.5 Gy XR (AD + CD)	127	230	261	130	187^*b*^
Expected values (AD + 1.0 Gy)	93	199	155	119	
% decrease (AD + 1.0 Gy)	11.8	7.5	1.3	7.6	
^*a*^*P-*value (AD + 1.0 Gy)	0.236	0.204	0.45	0.25	
Expected values (AD + 1.5 Gy)	138	212	243	151	
% decrease (AD + 1.5 Gy)	7.97	−8.49	−7.41	13.91	
^*a*^*P-*value (AD + 1.5 Gy)	0.273	0.81	0.80	0.09	
Untreated controls	13	13	9	4	10
1 ng/ml MMC (AD)	17	23	13	5	14
100 ng/ml MMC (CD)	30	50	45	39	41
1 ng/ml + 100 ng/ml MMC (AD + CD)	18	40	50	20	32
Expected values (AD + 100 ng/ml MMC)	34	60	49	40	
% decrease (AD + 100 ng/ml MMC)	47.1	33.3	−2.0	50	
^*a*^*P-*value (AD + 100 ng/ml MMC)	0.036	0.037	0.5	0.004	

Expected values in AD + CD were the sums of two individual treatments minus that of untreated controls. ^*a*^*P-*values are based on the one-tailed *z*-test. ^*b*^Paired two-tailed *t*-test for the difference between RF + 1.5 Gy XR/Sham + 1.5 Gy X-rays, *P* = 0.033.

The lymphocytes from donors exposed to 1 cGy XR alone (AD) did not show a significant increase in the incidence of MN as compared with those of unexposed controls. There was a differential response of the lymphocytes, among the donors, when 1 cGy XR was combined with 1.0 Gy or 1.5 Gy XR (AD + CD). (i) Lymphocytes from D2 and D4 exposed to 1 cGy + 1.0 Gy (AD + CD) showed significantly decreased MN, 47% and 21%, respectively (*P*<0.05), while cells from D1 and D3 did not show such a response and the decrease in MN was 8.4% and − 4.5%, respectively (*P*>0.05). (ii) Lymphocytes from all donors exposed to 1 cGy + 1.5 Gy (AD + CD) showed a significantly decreased incidence of MN that ranged from 23% to 69% (*P*<0.05).The lymphocytes from donors exposed to RF (AD) did not show a significant increase in the incidence of MN as compared with those of unexposed controls. There was a differential response between the four donors when their cells were exposed to RF combined with 1.0 Gy or 1.5 Gy XR (AD + CD). (i) Lymphocytes from D2 and D4 exposed to RF + 1.0 Gy XR (AD + CD) showed ∼30% decrease in the incidence of MN (*P*<0.05), while the cells from D1 and D3 did not show such a decrease (1% and − 10%, respectively) (*P*>0.05). (ii) Lymphocytes from D1, D2 and D4 exposed to RF + 1.5 Gy XR (AD + CD) showed a significantly decreased incidence of MN, ranging from 31–60% (*P*<0.05), while the cells from D3 did not show such a decrease in MN, (only 2.5%) (*P*>0.05). It is interesting to note that the cells from D1, which did not exhibit AR after 1.0 Gy XR did show AR after 1.5 Gy XR.

The lymphocytes from donors exposed to sham (AD) did not show a significant increase in the incidence of MN as compared with those of unexposed controls. In addition, there was no significant decrease in the incidence of MN in cells exposed to sham + 1.0 or 1.5 Gy XR (AD + CD), indicating that pre-sham exposure of the cells had no impact on the incidence of MN induced by subsequent exposure to high-dose XR.

The lymphocytes from donors exposed to 1 ng/ml MMC alone (AD) showed no significant increase in MN as compared with those of unexposed controls. Lymphocytes from D1, D2 and D4 treated with 1 ng/ml + 100 ng/ml MMC (AD + CD) showed a significantly decreased incidence of MN, i.e. 47%, 33% and 50%, respectively, (*P*<0.05), while such a decrease was not observed in donor D3 (*P*>0.05).

The data presented in Table 2 refer to PI. They show a statistically significant decrease in PI in cells exposed to 1.0 Gy or 1.5 Gy and 100 ng/ml MMC compared with untreated controls, indicating cytotoxicity induced by CD exposure (*P*<0.05). With respect to the cells exposed to AD + CD (1.0 cGy + 1.0 Gy, 1.0 cGy + 1.5 Gy, RF + 1.0 Gy, RF + 1.5 Gy, sham + 1.0 Gy, sham + 1.5 Gy, 1 + 100 MMC), the PI was similar to that in cells exposed to CD alone, and significantly lower than in untreated control (and sham-exposed) cells. Thus, overall the data suggest that RF-induced AR is not associated with significant changes in the rate of cell proliferation.

**Table 2.** Proliferation Index (1000 cells) in lymphocytes from four donors (D1–D4) exposed to 1.0 cGy ± 1.0 or 1.5 Gy XR and 1950 MHz RF/sham ± 1.0 or 1.5 Gy XR and to 1 ng/ml ± 100 ng/ml MMC

Treatment	Proliferation Index				
	D1	D2	D3	D4	Average
1.0 cGy	1.90	2.08	1.75	1.58	1.83
1.0 Gy	1.72	1.69	1.57	1.38	1.59*
1.5 Gy	1.65	1.80	1.41	1.30	1.54*
1.0 cGy + 1.0 Gy	1.56	1.69	1.57	1.52	1.59**
1.0 cGy + 1.5 Gy	1.65	1.80	1.49	1.38	1.58*
RF	1.84	1.96	1.76	1.78	1.84
RF + 1.0 Gy	1.73	1.70	1.48	1.46	1.59*
RF + 1.5 Gy	1.55	1.67	1.45	1.34	1.50**
Sham	1.75	1.95	1.70	1.84	1.81
Sham + 1.0 Gy	1.56	1.66	1.42	1.50	1.54***
Sham + 1.5 Gy	1.60	1.71	1.46	1.47	1.56***
Un-treated controls	1.89	2.10	1.96	1.82	1.94
1 ng/ml MMC	1.86	2.11	1.80	1.66	1.86
100 ng/ml MMC	1.81	1.69	1.64	1.54	1.67*
1 + 100 MMC	1.63	1.74	1.64	1.54	1.64**

Paired two-tailed *t*-test. *P<0.05; **P<0.005 (vs untreated controls); ^***^P<0.005 (vs sham).

## DISCUSSION

There is well-documented scientific evidence for the existence of very low-dose ionizing radiation-induced AR in human blood lymphocytes. Such AR has been further characterized and putatively linked to DNA-repair processes and *de novo* protein synthesis [1–14]. The data from our earlier studies indicated that non-ionizing RF is also capable of inducing AR. Human blood lymphocytes obtained from several donors were stimulated with PHA for 24 h and then exposed to 900 MHz RF (GSM signal, 1.25 W/kg average SAR) for 20 h (AD). This was followed by treatment of the cells with a genotoxic dose of 100 ng/ml MMC (CD). The incidence of MN was found to be significantly decreased in the cells exposed to AD + CD as compared with those exposed to CD alone, thus indicating RF-induced AR to the subsequent exposure to MMC. The data also indicated variability among the donors in their response to AD + CD exposures: some donors exhibited AR while others did not [17]. These observations were confirmed in two subsequent investigations, which characterized the RF-induced AR further to indicate the impact of: (i) cell cycle (cells exposed in S-phase of the cell cycle exhibited RF-induced AR while those in G0 and G1 phase did not) [18], and (ii) SAR (pre-exposure of S-phase cells to 1950 MHz RF (UMTS signal) exhibited varying degrees of RF-induced AR, with maximum decrease in MN at an average SAR of 0.3 W/kg, a lesser decrease at 0.6 W/kg SAR, and no decrease at 0.15 W/kg or 1.25 W/kg) [19]. Thus, the overall data indicated that the extent of RF-induced AR was variable and depended on the stage at which the cells were pre-exposed to RF and on the electromagnetic characteristics of RF exposure.

RF-induced AR was also reported in experimental mice that were pre-exposed to 900 MHz RF (120 µW/cm^2^, corresponding to 55 mW/kg) and then subjected to gamma-radiation (CD). A significantly increased survival, significantly decreased hematopoietic tissue damage, increased expression of cell cycle-related *cyclin-D1*, *cyclin-E*, *cyclin-DK4* and *cyclin-DK2* genes and decreased primary DNA damage in blood leukocytes were observed in mice exposed to AD + CD as compared with mice exposed to CD alone [20–22]. The RF-induced AR was also confirmed in independent investigations using experimental mice and rats, although no details on electromagnetic dosimetry were given [24, 25]. Furthermore, *in vitro* studies on human promyelocytic leukemia HL-60 cells that were pre-exposed to 900 MHz RF (0.25 µW/kg average SAR) and then exposed to the chemotherapeutic drug doxorubicin showed increased cell proliferation, decreased apoptosis, increased mitochondrial membrane potential, decreased intracellular Ca^2+^ and increased Ca^2+^-Mg^2+^-ATPase activity [23]. Together these observations provide some mechanistic insights into RF-induced AR in experimental animals and in HL-60 cells.

As mentioned above, in our previous investigations, we have used RF pre-exposure as AD and subsequent treatment with a genotoxic dose of MMC as CD to demonstrate the induction of AR in human blood lymphocytes [17–19]. MMC is a chemical mutagen. It is an extensively used chemotherapeutic drug and is widely reported to induce cross-links between the complementary strands in DNA by reductive activation followed by two N-alkylations that are sequence-specific for guanine nucleoside in the sequence 5′-CpG-3′ [34–36]. We considered it important to investigate whether or not RF pre-exposure can offer protection to cells in order to provide resistance to damage induced by other kinds of lesions in the DNA and thus induce AR. In this preliminary investigation, we chose to use XR as CD. It is well known that the predominant indirect action of XR involves its interaction with water molecules in the cell to produce free radicals that diffuse far enough to reach the DNA, inducing strand breaks [37].

Our results indicated a significantly decreased incidence of MN in RF pre-exposed cells when they were subsequently exposed to XR, thus non-ionizing RF induced AR to ionizing XR. Furthermore, the observations made in this preliminary study also indicated that the cells exposed to RF + XR (AD + CD) did not show significant changes in PI, while those exposed to XR and MMC alone (CD) showed a significant decrease in PI. These data further suggested that RF-induced AR was not related to alteration in cell proliferation.

There is a large body of information in the peer-reviewed scientific literature concerning the mechanistic aspects of low-dose ionizing-radiation-induced AR [38–44]. So far, very little is known regarding the mechanism(s) of RF-induced AR. Our further research will focus on the mechanism(s) as well as on the examination of variability in RF-induced AR in the lymphocytes obtained from a range of donors. The beneficial effect of RF pre-exposure that has been observed in the study and in the other studies mentioned above is worthy of special attention.

## FUNDING

This work has been supported by National Institute of Nuclear Physics (INFN), section of Naples, Project ARCAICA.
